# Involvement of 5-lipoxygenase activating protein in the amyloidotic phenotype of an Alzheimer’s disease mouse model

**DOI:** 10.1186/1742-2094-9-127

**Published:** 2012-06-14

**Authors:** Jin Chu, Domenico Praticò

**Affiliations:** 1 Center for Translational Medicine, Department of Pharmacology, Temple University School of Medicine, 3420 North Broad Street MRB, 706A, Philadelphia, PA, 19140, USA

**Keywords:** Alzheimer’s disease, Amyloid β, Amyloid beta precursor protein, Animal model, 5-lipoxygenase activating protein

## Abstract

**Background:**

The 5-lipoxygenase enzyme is widely distributed within the central nervous system and its activity is regulated by the presence and availability of another protein, called 5-lipoxygenase activating protein. While previous works have shown that 5-lipoxygenase is involved in the pathogenesis of Alzheimer’s disease, no data are available on the role that 5-lipoxygenase activating protein plays in Alzheimer’s disease.

**Methods:**

In the present paper, we studied the effect of pharmacologic inhibition of 5-lipoxygenase activating protein on the amyloidotic phenotype of Tg2576 mice.

**Results:**

Amyloid β peptide (Aβ) deposition in the brains of mice receiving MK-591, a selective and specific 5-lipoxygenase activating protein inhibitor, was significantly reduced when compared with controls. This reduction was associated with a similar decrease in brain Aβ peptides levels. MK-591 treatment did not induce any change in the steady-state levels of amyloid-β precursor protein, β-site amyloid precursor protein cleaving enzyme 1 or disintegrin and metalloproteinase domain-containing protein 10. By contrast, it resulted in a significant reduction of the γ-secretase complex, at the protein and message level. Furthermore, *in vitro* studies confirmed that MK-591 prevents Aβ formation by modulating γ-secretase complex levels without affecting Notch signaling.

**Conclusions:**

These data establish a novel functional role for 5-lipoxygenase activating protein in the pathogenesis of Alzheimer’s disease-like amyloidosis, and suggest that its pharmacological inhibition could provide a novel therapeutic opportunity for Alzheimer’s disease.

## Background

The enzyme 5-lipoxygenase (5LO) catalyzes the conversion of arachidonic acid to 5-hydroxyperoxyeicosatetraenoic acid and subsequently to 5-hydroxyeicosatetraenoic acid, which can be then metabolized into different leukotrienes [1]. It is abundantly present in the central nervous system (CNS), where its activity is regulated by the presence and availability of another protein, 5LO-activating protein (FLAP) [2]. From a biochemical point of view, they form a functional complex whose integrity is necessary for the full 5LO enzymatic activity. A peculiar aspect of the FLAP/5LO pathway is the fact that its expression levels are significantly increased in the CNS with aging, and that this increase is also region-specific since it mainly manifests in the hippocampus, an area vulnerable to neurodegenerative insults [3].

Interestingly, recent studies showed that hippocampi from patients with Alzheimer’s disease (AD) have higher 5LO immunoreactivity when compared with healthy controls, and that the genetic absence of 5LO results in a significant reduction of the brain amyloidotic phenotype of amyloid-β precursor protein (APP) transgenic mice (that is, Tg2576) [4,5]. Taken together, these data suggest an involvement of this pathway in AD pathogenesis, and support the hypothesis that it plays a functional role in AD development.

However, no studies are available testing the specific and direct role that FLAP may also play in the development of the AD-like amyloidotic phenotype of the Tg2576 mice.

To this end, in the current study we chronically administered these mice with a selective and specific orally available inhibitor of FLAP activation, MK-591 [6]. At the end of the study, compared with mice receiving vehicle, the group treated with MK-591 showed a significant reduction in the amount of amyloid β peptide (Aβ) formed and deposited in their brains. These changes were not associated with any significant modification of total APP, β-site amyloid precursor protein cleaving enzyme 1 (BACE-1) or disintegrin and metalloproteinase domain-containing protein 10 (ADAM-10) protein levels. By contrast, we observed that the group administered with MK-591 had a significant reduction of the γ-secretase complex at the protein and message level. These results were further confirmed *in vitro* using neuronal cells stably expressing the human APP Swedish mutant, neuro-2 A neuroblastoma (N2A)-APPswe cells.

## Methods

### Mice and treatments

All animal procedures were approved by the Institutional Animal Care and Usage Committee and in accordance with the National Institute of Health guidelines. The Tg2576 transgenic mice expressing human APP with the Swedish mutation (K670N/M671L) used in these studies were as previously described [7]. They were genotyped by PCR analysis using tail DNA and kept in a pathogen-free environment, on a 12-hour light/dark cycle and had access to food and water *ad libitum*. All the experiments presented in this paper were performed with female mice. Starting at 7 months of age, mice were randomized to receive MK-591 (40 mg/kg weight) (n = 11) or vehicle (n = 9) in their chow diet for 8 months until they were 15 months old. Considering that each mouse eats on average 5 g/day of chow diet and the diet is formulated for 320 mg MK-591 per kg diet (Harlan Teklad, WI, USA), the final dose of the active drug was approximately 40 mg/kg weight/day. During the study, mice in both groups gained weight regularly, and no significant difference in weight was detected between the two groups. No macroscopic effect on the overall general health was observed in the animals receiving the active treatment. Post-mortem examination showed no sign of macroscopic pathology in any of the organs considered (spleen, liver, thymus, ileum).

After sacrifice, animals were perfused with ice-cold 0.9% PBS, the brain removed and dissected in two by midsagittal dissection. One was immediately stored at −80°C for biochemistry assays or total RNA extraction, and the other immediately immersed in 4% paraformaldehyde in 0.1 M PBS (pH 7.6) overnight for immunohistochemistry studies.

### Immunohistochemistry

Immunostaining was performed as reported previously by our group [8,9]. Serial 6-μm-thick coronal sections were mounted on 3-aminopropyltriethoxysilane-coated slides. Every eighth section from the habenular to the posterior commissure (8 to 10 sections per animal) was examined using unbiased stereological principles. The sections for Aβ were deparaffinized, hydrated, pretreated with formic acid (FA; 88%) and subsequently with 3% hydrogen peroxide in methanol. The sections for glial acidic fibrillary protein (GFAP) and CD45 were deparaffinized, hydrated and treated with 3% hydrogen peroxide in methanol and subsequently antigen retrieved with citrate (10 mM). Sections were blocked in 2% fetal bovine serum before incubation with primary antibodies (4 G8 for Aβ, anti-GFAP, and anti-CD45) overnight at 4°C. Subsequently, sections were incubated with biotinylated anti-mouse IgG (Vector Laboratories, Burlingame, CA, USA) and then developed using the avidin-biotin complex method (Vector Laboratories) with 3,3’-diaminobenzidine as a chromogen. Light microscopic images were used to calculate the area occupied by Aβ-immunoreactivity using the software Image-Pro Plus for Windows version 5.0 (Media Cybernetics, Bethesda, MD, USA). The threshold optical density that discriminated staining from background was determined and kept constant for all quantifications. The area occupied by Aβ-immunoreactivity was measured by the software and divided by the total area of interest to obtain the percentage area of Aβ-immunoreactivity.

### Biochemical analyses

Mouse brain homogenates were sequentially extracted first in radioimmunoprecipitation assay (RIPA) for the Aβ soluble fractions and then in FA for the Aβ insoluble fractions as previously described [5,8,9]. Aβ1-40 and Aβ1-42 levels were assayed by a sensitive sandwich ELISA kits (WAKO Chem, Richmond, VA, USA.). Interleukin 1-β (IL-1β) levels in brain homogenates were assayed by a specific and sensitive sandwich ELISA kit, following the manufacturer’s instructions (R&D Systems, Minneapolis, MN, USA). Supernatants from the cell culture experiments were also assayed for their levels of lactate dehydrogenase (LDH) by a colorimetric assay kit (BioVision, Milpitas, CA, USA). Analyses were always performed in duplicate and in a coded fashion.

### Western blot analyses

RIPA extracts from brain homogenates were used for western blot analyses. Samples were electrophoresed on 10% Bis-Tris gels or 3% to 8% Tris-acetate gel (Bio-Rad, Richmond, CA, USA), according to the molecular weight of the target molecule, transferred onto nitrocellulose membranes (Bio-Rad), and then incubated with appropriate primary antibodies as follows: anti-APP N-terminal raised against amino acids 66 to 81 for total APP (22 C11, Chemicon International, Temecula, CA, USA), anti-BACE-1 (IBL America, Minneapolis, MN,USA), anti-ADAM-10 (Chemicon), anti-secreted-APPα (2B3, IBL America), anti-secreted-APPβ (6A1, IBL America), anti-C-terminal fragments (CTF; EMD Biosciences, Inc., Billerica, MA, USA), anti-presenilin1 (PS1; Sigma, St. Lousi, MO,USA), anti-nicastrin (Cell Signaling, Daners, MA, USA), anti-anterior pharynx-defective 1 (APH-1; Millipore, Billerica, MA, USA), anti-presenilin enhancer 2 (Pen-2; Invitrogen, Grand Island, NY, USA), anti-GFAP (Santa Cruz Biotechnology, Santa Cruz, CA, USA), anti-5LO (BD Transduction Laboratories, San Jose, CA, USA); anti-Notch intracellular domain (NICD; Cell Signaling), anti-insulin-degrading enzyme (IDE) N-terminal (EMD Biosciences), anti-neprilysin (Santa Cruz Biotechnology, Inc.), anti- apolipoprotein E (Santa Cruz Biotechnology, Inc.), anti-cAMP response element-binding protein (CREB; Cell Signaling) and anti-phosphorylated-CREB (Cell Signaling), anti-Sp1 (Santa Cruz Biotechnology, Inc.) and anti-β actin (Santa Cruz Biotechnology, Inc.).

After three washings with T-TBS (Tween-Tris Buffered Saline), membranes were incubated with IRDye 800CW or IRDye 680CW-labeled secondary antibodies (LI-COR Bioscience, Lincoln, NE, USA) at 22°C for 1 h. Signals were developed with Odyssey Infrared Imaging Systems (LI-COR Bioscience). β-actin was always used as internal loading control.

### Real-time quantitative RT-PCR amplification

RNA was extracted and purified using the RNeasy mini-kit (Qiagen, Valencia, CA, USA), as previously described [10]. Briefly, 1 μg of total RNA was used to synthesize cDNA in a 20 μL reaction using the RT²First Strand Kit RT-PCR (Super Array Bioscience, Valencia, CA, USA). Mouse BACE-1, PS1, nicastrin, APH-1 and Pen-2 genes were amplified by using the corresponding primers designed and synthesized by Super Array Bioscience. β-actin was always used as an internal control gene to normalize for the amount of RNA. Real-time PCR was performed in an Eppendorf ep realplex thermal cycler (Eppendorf, Hauppauge, NY, USA). Two microliters of cDNA was added to 25 μL of SYBR Green PCR Master Mix (Applied Biosystems, Carlsbad, CA, USA). Each sample was run in duplicate, and analysis of relative gene expression was done by using the 2^-ΔΔCt^ method [11]. Briefly, the relative change in gene expression was calculated by subtracting the threshold cycle (ΔCt) of the target genes from the internal control gene (β-Actin). Based on the fact that the amount of cDNA doubles in each PCR cycle (assuming a PCR efficiency of 100%), the final fold-change in gene expression was calculated by using the formula relative change = 2^-ΔΔCt^[11].

### Cell cultures

N2A cells stably expressing human APP carrying the K670N/M671L Swedish mutation (APPswe) were grown in Dulbecco’s modified Eagle medium supplemented with 10% fetal bovine serum, 100 U/mL penicillin, 100 μg/mL streptomycin (Cellgro, Herdon, VA, USA), and 400 μg/mL G418 (Invitrogen), at 37°C in the presence of 5% CO_2_ as previously described [10].

For each experiment, equal numbers of cells were plated in six-well plates; 24 h later media were removed and fresh media containing either MK-591 (1 μM, 10 μM or 25 μM) or vehicle were added. After incubation for 24 h, supernatants were collected for Aβ and LDH measurement, and cell pellets harvested in lytic buffer for immunoblot analyses as described in the previous paragraphs.

For transfection studies, N2A-APPswe cells were transfected with 1 μg Myc-tagged mΔE-Notch-1 complementary DNA overnight (a generous gift from Dr. L. D´Adamio, Albert Einstein Medical College, NY, USA) by using Lipofectamine 2000 (Invitrogen). The media were removed and fresh media containing MK-591, L685,458 or vehicle were added. After incubation for 24 h, cells lysates were collected NICD expression levels assayed by western blot analysis.

### Data analysis

Data analyses were performed using SigmaStat for Windows version 3.00. Statistical comparisons were performed by unpaired Student’s *t*-test or the Mann–Whitney rank sum test when a normal distribution could not be assumed. Values represent mean ± standard error of the mean. Significance was set at *P* <0.05.

## Results

### *In vivo* studies

#### 5-lipoxygenase activating protein blockade reduces brain Aβ peptides levels and deposition

Starting at 7 months of age, Tg2576 mice were randomized to receive MK-591 (320 mg/kg diet) or vehicle in their chow diet for 8 months before being killed. Considering that each mouse eats on average 5 g/day of chow diet, the final dose of the active drug was approximately 40 mg/kg weight/day. By the end of the study, body weight, total plasma cholesterol, triglycerides and blood cell counts were not different between the two groups (not shown).

**Figure 1:**
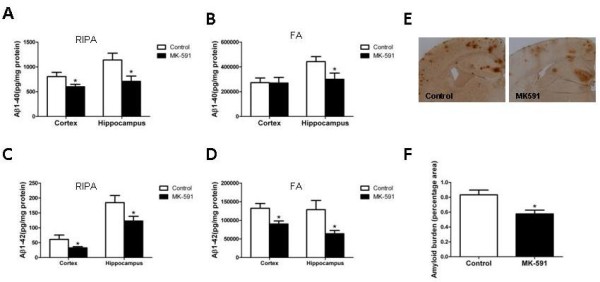
**Pharmacologic blockade of 5-lipoxygenase activating protein decreases brain Aβ peptides levels and deposition. (A-D)** RIPA-soluble (RIPA) and formic acid extractable (FA) Aβ1-40 and Aβ1-42 levels in cortex and hippocampus of Tg2576 receiving MK-591 or placebo for 8 months were measured by sandwich ELISA. (n = 9 for control, and n = 11 for MK-591; *P <0.04). **(E)** Representative sections of brains from Tg2576 mice receiving MK-591 or placebo (control) for 8 months immunostained with 4 G8 antibody. **(F)** Quantification of the area occupied by Aβ immunoreactivity in brain of Tg2576 mice receiving MK-591 or placebo for 8 months (*P = 0.03). ELISA: enzyme-linked immunosorbent assay; FA: formic acid; RIPA: radioimmunoprecipitation assay.

As expected for their age, 15-month-old Tg2576 mice on vehicle showed elevated levels of both soluble (RIPA extractable) and insoluble (FA extractable) Aβ1-40 and Aβ1-42 in their cerebral cortex as well as hippocampus, the levels of which were significantly reduced in mice receiving MK-591 (Figure 1A-D).

Amyloid deposits were widely distributed in the cerebral cortex and hippocampus of Tg2576 mice at 15 months of age, as previously reported [12]. To determine the effect of chronic MK-591 administration on brain amyloid deposition, the areas occupied by 4 G8-immunopositive reactions were analyzed. Comparison of the Aβ-immunopositive areas between the placebo and MK-591-treated group revealed a statistically significant reduction of the amyloid burden in the treated mice (Figure 1E, F).

#### 5-lipoxygenase activating protein blockade influences brain amyloid-β precursor protein metabolism

**Figure 2:**
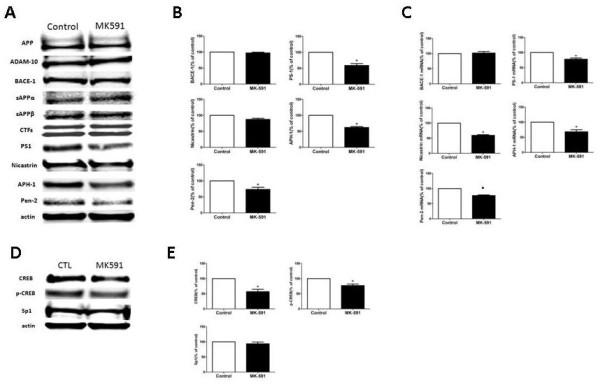
**Pharmacologic blockade of 5-lipoxygenase activating protein alters amyloid-β precursor protein metabolism via the γ-secretase pathway. (A)** Representative western blots of APP, ADAM-10, BACE-1, secreted APPα, secreted APPβ, CTFs, PS1, nicastrin, APH-1 and Pen-2 in brain homogenates of Tg2576 mice receiving MK-591 or placebo for 8 months. **(B)** Densitometric analysis of some of the immunoreactivities to the antibodies shown in the previous panel (n = 9 control, n = 11 MK-591; *P <0.04). **(C)** Relative mRNA levels for BACE-1, PS1, nicastrin, APH-1 and Pen-2 in brain homogenates of Tg2576 mice receiving MK-591 or placebo for 8 months, as determined by real-time quantitative RT-PCR amplification (*P <0.02). **(D)** Representative western blot for total cAMP response element-binding and its phosphorylated form at Ser133, and Sp1 in brain homogenates of Tg2576 mice receiving MK-591 or placebo. **(E)** Densitometric analysis of the immunoreactivities to the antibodies shown in the previous panel (n = 9 control, n = 11 MK-591; *P <0.03). ADAM-10: disintegrin and metalloproteinase domain-containing protein 10; APH-1: anterior pharynx-defective 1; APP: amyloid-β precursor protein; BACE-1: β-site amyloid precursor protein cleaving enzyme 1; CREB: cAMP response element-binding protein; CTF: C-terminal fragments; Pen-2: presenilin enhancer 2; PS1: presenilin1; RT-PCR: reverse transcriptase polymerase chain reaction.

Since Aβ is the final product of the proteolytic processing of its own precursor, APP, next we investigated whether this pharmacologic treatment was associated with an alteration on the expression levels of this protein. As shown in Figure 2A, we found that there was no difference in total APP levels between the two groups of mice. To assess the effect of MK-591 on APP processing, we investigated the steady-state levels of the main enzyme proteases involved, α-secretase (ADAM-10), β-secretase (BACE-1) and the four components of the γ-secretase complex, by western blot analysis. As shown in Figure 2A, B, no significant differences in the levels of ADAM-10, BACE-1, secreted APPα, secreted APPβ or CTFs were observed between the two groups of mice. By contrast, we observed that mice receiving MK-591 had a statistically significant reduction in the steady-state levels of three of the four components of the γ-secretase complex, PS1, Pen-2 and APH-1 (Figure 2A, B). Despite an observed reduction for nicastrin, it did not reach statistical significance (p = 0.06). Furthermore, quantitative real-time RT-PCR analyses revealed that, in the same mice, the mRNA levels of these four proteins were significantly reduced (Figure 2C). By contrast, no change was observed in the BACE-1 mRNA levels (Figure 2C).

#### 5-lipoxygenase activating protein blockade does not affect Aβ catabolic pathways

Since the final amount of Aβ assayed is the result of production and degradation, next we analyzed two of the major proteases involved in its catabolism: IDE and neprilysin [13]. Steady-state levels of both proteins measured by western blot analysis were similar between the two groups of mice (not shown). A similar result was observed when we measured levels of the apolipoprotein E, which has been implicated in the clearance of Aβ from the CNS by acting as chaperone [14] (not shown).

#### 5-lipoxygenase activating protein blockade modulates neuroinflammation

**Figure 3:**
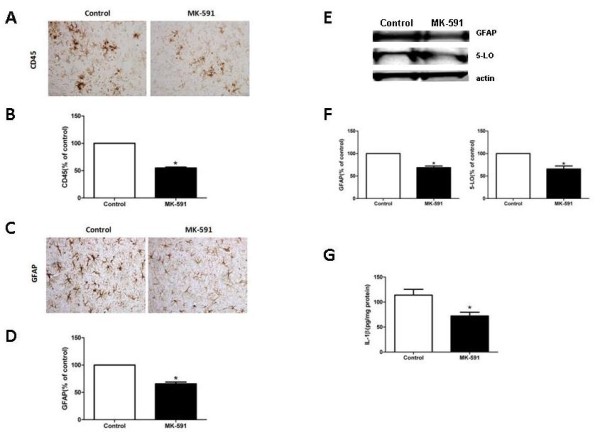
**Pharmacologic blockade of 5-lipoxygenase activating protein reduces neuroinflammation. (A, C)** Representative brain sections of the cortex region from Tg2576 receiving MK-591 or vehicle (control) immunostained for CD45 and GFAP (× 20 magnification)**. (B, D)** Quantitative analysis of the immunoreactivity for CD45 and GFAP in the same animals. **(E)** Representative western blot analysis of GFAP and 5-LO in brain homogenates of Tg2576 receiving MK-591 or control. **(F)** Densitometric analysis of the immunoreactivities to the antibodies shown in the previous panel. **(G)** Levels of IL-1β in brain homogenates of Tg2576 mice receiving MK-591 or controls (n = 8 controls, n = 10 MK-591;*P = 0.01). CD45: cluster of differentiation 45; GFAP: glial acidic fibrillary protein; 5-LO: 5-lipoxygenase.

Since neuroinflammation is also an important feature of this AD-like amyloidosis model [15], next we investigated the effect of FLAP pharmacologic blockade on microglia and astrocytes activation. As shown in Figure 3A-D, mice receiving MK-591 had a significant decrease in the immunoreactivity for CD45, a marker of microgliosis, and GFAP, a marker of astrogliosis. Immunoblot analysis confirmed the significant reduction of GFAP in brain homogenates from mice receiving MK-591, which was accompanied by a significant reduction in the steady-state levels of 5-LO (Figure 3E, F). Finally, we observed that animal receiving MK-591 also had a significant reduction in brain levels of IL-1β (Figure 3G).

#### MK-591 affects cAMP response element-binding protein but not Sp1

The data collected so far suggest that MK-591 by blocking FLAP regulates the γ-secretase complex expression at the transcriptional level. Since previous studies have shown that 5LO activation by producing hydroxy-eicosatetraenoic acids can influence CREB, a transcriptional factor that regulates gene expression [10], we wanted to test if this was also the case in our system. Compared with mice on placebo, we found that mice treated with MK-591 showed a statistically significant decrease in the steady-state levels of total CREB and its phosphorylated form at Ser133. However, MK-591 did not significantly affect the steady-state levels of Sp1, another transcription factor (Figure 2D, E).

### *In vitro* studies

#### MK-591 influences Aβ formation in a γ-secretase-dependent manner

**Figure 4:**
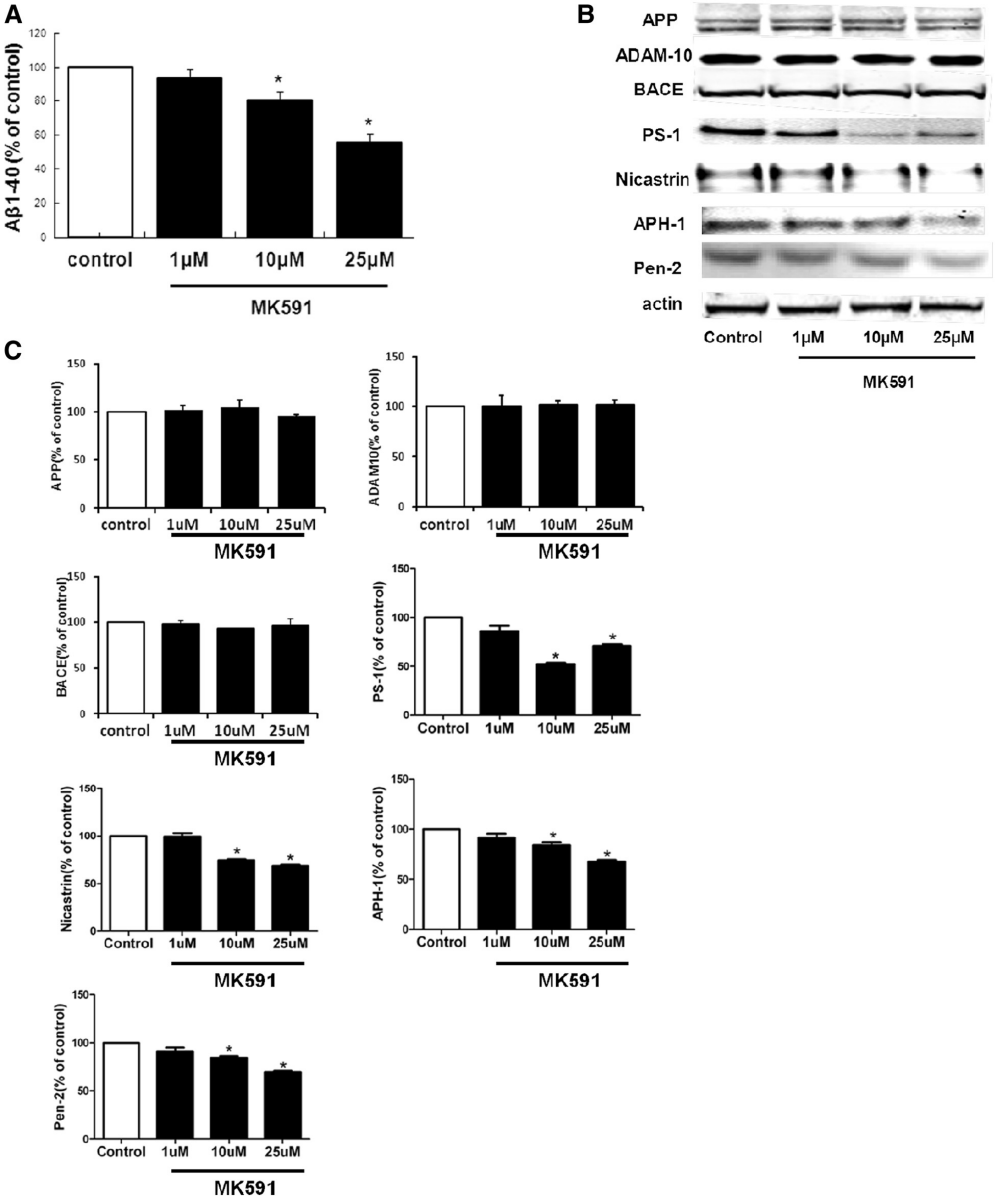
***In vitro*effect of MK-591 on Aβ formation and amyloid-β precursor protein metabolism.** N2A-APPswe cells were incubated with increasing concentration of MK-591 or vehicle for 24 h, and conditioned media and cell lysates collected. **(A)** Aβ1-40 levels in the supernatant assayed by sandwich ELISA (n = 4 per each condition; *P <0.01). **(B)** Representative western blots of APP, ADAM-10, BACE-1, PS1, nicastrin, APH-1, and Pen-2 in the lysates of MK-591 or vehicle-treated cells. **(C)** Densitometric analyses of the immunoreactivities to the antibodies shown in panel B (*P <0.01). ADAM-10: disintegrin and metalloproteinase domain-containing protein 10; APH-1: anterior pharynx-defective 1; APP: amyloid-β precursor protein; BACE-1: β-site amyloid precursor protein cleaving enzyme 1; ELISA: enzyme-linked immunosorbent assay; N2A-APPswe: neuro-2 A neuroblastoma cells expressing human APP carrying the K670N/M671L Swedish mutation; Pen-2: presenilin enhancer 2; PS1: presenilin1.

To further confirm our *ex vivo* observation, we embarked in a series of *in vitro* experiments. N2A-APPswe cells were incubated with MK-591 for 24 hours at different concentration (1 μM, 10 μM and 25 μM) or vehicle. At the end of this period, conditioned media showed that, compared with control (0.327 ± 0.003), the presence of MK-591 did not alter the levels of LDH (1 μM, 0.347 ± 0.01; 10 μM, 0.331 ± 0.01; 25 μM, 0.346 ± 0.006; absorbance 450 nm). The same drug reduced Aβ1-40 formed by these cells in a dose-dependent manner (Figure 4A). This reduction was associated with a significant decrease in the steady-state levels of PS1, nicastrin, APH-1 and Pen-2 proteins, the four components of the γ-secretase complex (Figure 4B, C). By contrast, MK-591 did not influence the protein levels for APP, BACE-1 or ADAM-10 (Figure 4B, C).

#### MK-591 influences cAMP response element-binding protein but not Sp1

**Figure 5:**
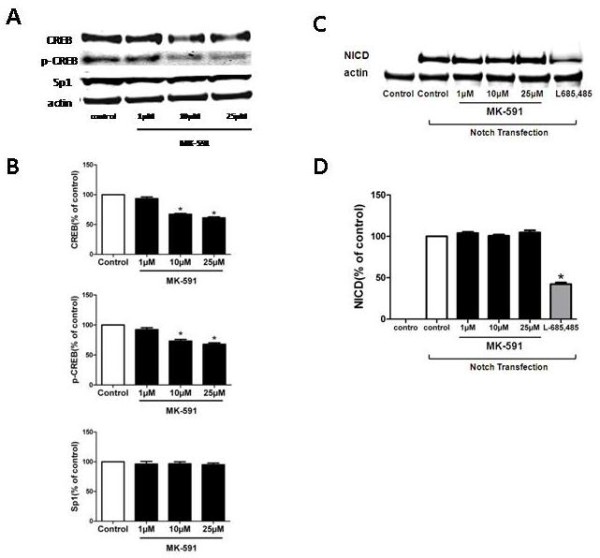
***In vitro*effect of MK-591 on cAMP response element-binding protein and Notch.** N2A-APPswe cells were incubated with increasing concentration of MK-591 or vehicle for 24 hours, and cell lysates collected for immunoassays. **(A)** Representative western blots of total CREB, its phosphorylated form (p-CREB) at Ser133, and Sp1 levels. **(B)** Densitometric analyses of the immunoreactivities to the antibodies to CREB, p-CREB and Sp1 (*P <0.01). **(C)** Representative western blots of NICD in the lysates of cells treated with MK-591, L685,458 or vehicle groups. **(D)** Densitometric analyses of the immunoreactivities to the antibodies NICD (n = 4; *P <0.01). CREB: cAMP response element-binding protein; NICD: Notch intracellular domain; N2A-APPswe: neuro-2 A neuroblastoma cells expressing human APP carrying the K670N/M671L Swedish mutation.

Similar to the *in vivo* experiments, we also observed that incubation of MK-591 with N2A-APP cells resulted in a significant decrease in the expression levels of CREB and p-CREB. By contrast, the presence of the drug did not induce any significant alteration in the levels of the transcription factor Sp1 (Figure 5A, B).

#### MK-591 does not affect Notch signaling

Since Notch is another possible substrate for γ-secretase proteolytic activity, we tested whether this pathway was affected by the treatment. To this end, we assessed the effect of MK-591 on γ-secretase-mediated cleavages of Notch. N2A-APPswe cells were transfected with Myc-tagged mΔE-Notch-1 cDNA and incubated with MK-591 at the same concentrations which reduced γ-secretase components. We then assessed the expression levels of NICD by western blot analysis. As shown in Figure 5C, D no significant difference in the levels of NICD was observed between cells with and without MK-591 treatment. By contrast, when the specific γ-secretase inhibitor L685,458 was used, a significant reduction in NICD levels was detected (Figure 5C, D).

## Discussion

The data presented in this study demonstrate that pharmacologic blockade of FLAP significantly reduces brain Aβ formation and deposition in the Tg2576 mouse model of AD, and thereby provide the first evidence that this protein is a novel therapeutic target for modulating amyloidogenesis *in vivo*.

FLAP is an integral membrane protein of 18 kDa with the known function of activating the 5LO enzyme by directly associating and presenting it with its natural substrate, arachidonic acid, for the formation of potent biologically active lipids such as leukotrienes [16,17]. From a biochemical point of view, FLAP and 5LO form a functional complex whose integrity is necessary for the full enzymatic activation of this pathway [18].

Interestingly, in recent years a lot of work has been focused on the enzyme 5LO and its relationship with brain aging and AD-like amyloidosis. Thus, the 5LO protein is up-regulated in the CNS with aging, and its genetic deficiency or pharmacologic blockade significantly reduced brain amyloidosis in Tg2576 mice, suggesting a functional role for it in modulating Aβ levels and deposition [3,5,19]. However, so far no data are available on the specific and direct role that FLAP may have *in vivo* in the same transgenic mouse model.

With the present study, we wanted to test the hypothesis that the FLAP protein, alongside 5LO, is a potential target for modulating *in vivo* the AD-like brain amyloidotic phenotype of Tg2576 mice.

MK-591 is an orally available selective and specific FLAP inhibitor whose binding site partially overlaps with the arachidonic acid binding site, making it impossible for this substrate to be oxygenated by 5-LO [20]. We demonstrated that use of this inhibitor is associated with a significant reduction in brain amyloidosis in Tg2576 mice. In an effort to elucidate the mechanisms responsible for the Aβ reduction in the mice receiving MK-591, we assessed the steady state levels of APP and the levels of the three most important proteases involved in its processing, α-, β- and γ-secretase, which ultimately result in the formation of Aβ peptides. We found that total APP, BACE-1 (β-secretase) and ADAM-10 (α-secretase) protein levels were unaltered by the drug, suggesting that the *in vivo* biological effects of MK-591 are not mediated by modulation of the Aβ precursor or the α- or β-cleavage proteolytic pathways. By contrast, we observed that the γ-secretase complex was significantly reduced by this treatment, supporting the hypothesis that this pathway is specifically influenced by the active treatment. Interestingly, in association with a reduction in the steady-state levels of its proteins, we observed that their mRNAs were significantly decreased. This suggests translational regulation of the genes codifying for the γ-secretase complex.

Different potential transcriptional factor binding sites have been reported within the promoter regions of these genes. Among them, a CREB binding site has been shown to be essential for APH-1, Pen-2 and PS1 transcriptional regulation [21,22]. In our studies we found that, compared with the vehicle group, mice receiving MK-591 had a significant reduction in the levels of this transcription factor. By contrast, no difference between the two groups was observed when levels of Sp1, a transcription factor known to be involved in the regulation of the β-secretase-1 mRNA [23], were assayed, suggesting a specific effect on CREB.

Taken together, these findings support the hypothesis that FLAP pharmacological blockade by preventing 5LO activation modulates CREB levels and results in reduced transcription of the mRNAs for the γ-secretase complex, which ultimately is responsible for the reduction in Aβ formation *in vivo*.

Aβ is a major component of the hallmark AD brain lesions, and is generated from the sequential proteolytic processing of APP by the enzymes β- and γ-secretase [24]. The necessary role of γ-secretase in the pathogenesis of AD makes it a major target for drug development, with an effort focused on the ability to inhibit this complex. However, a key factor in establishing the clinical validity of γ-secretase inhibitors is the demonstration of a differential effect between APP processing and alternative substrates of this secretase, particularly the Notch signaling. Here, we demonstrate that the MK-591 effect on the γ-secretase complex is completely independent of any effect on the Notch signaling pathway. This *in vitro* observation is further supported by the fact that, during the study and at the end of the chronic treatment, animals receiving the active drug did not have any macroscopic difference in organs that are typical Notch targets.

The novel biological effect of the drug used in the current study is in agreement with previous studies and in line with a modulator activity of this drug on the secretase. It supports the novel idea that it is possible to develop γ-secretase modulators that alter Aβ formation while preserving other important functions of the complex [25]. This observation makes any potential therapeutic application of FLAP inhibitor(s), which could act as γ-secretase modulators, in AD feasible without the potential toxicity of the classical inhibitors of the complex [26,27].

## Conclusions

Our studies establish FLAP as a novel therapeutic target for AD-like amyloidosis. They represent the successful completion of the initial step for pre-clinical development of inhibitors of this protein as potential novel disease-modifying agents for AD.

## Abbreviations

Aβ, amyloid β peptide; AD, Alzheimer’s disease; APH-1, anterior pharynx-defective 1; APP, amyloid β precursor protein; BACE-1, β-site amyloid precursor protein cleaving enzyme 1; CNS, central nervous system; CREB, cAMP response element-binding protein; CTFs, C-terminal fragments; ELISA, enzyme-linked immunosorbent assay; FA, formic acid; FLAP, 5-lipoxygenase activating protein; GFAP, glial acidic fibrillary protein; IDE, insulin-degrading enzyme; Ig, immunoglobulin; IL, interleukin; kDa, kiloDalton; LDH, lactate dehydrogenase; NICD, Notch intracellular domain; N2A, neuro-2 A neuroblastoma; PBS, phosphate buffered saline; PCR, polymerase chain reaction; Pen-2, presenilin enhancer 2; PS1, presenilin1; RIPA, radioimmunoprecipitation assay; RT, reverse transcriptase; Tg2576 mice, transgenic mice over-expressing human Swedish mutant of APP; 5LO, 5-lipoxygenase.

## Competing interests

The authors declare that they have no competing interests.

## Authors' contributions

JC and DP designed the study; JC performed the *in vivo* and *in vitro* experiments, collected and analyzed the data and drafted the manuscript; DP analyzed the data and wrote the manuscript. All authors read and approved the final manuscript.

## References

[B1] Murphy RC Gijon MA Biosynthesis and metabolism of leukotrienes Biochem J 2007 405 379 395 10.1042/BJ20070289 17623009

[B2] Mandal AK Skoch J Bacskai BJ Hyman BT Christmas P Miller D Yamin TT Xu S Wisniewski D Evans JF Soberman RJ The membrane organization of leukotriene synthesis Proc Natl Acad Sci USA 2004 101 6587 6592 10.1073/pnas.0308523101 15084748 PMC404089

[B3] Chinnici CM Yao Y Praticò D The 5-lipoxygenase enzymatic pathway in the mouse brain: young versus old Neurobiol Aging 2007 28 1457 1462 10.1016/j.neurobiolaging.2006.06.007 16930777

[B4] Ikonomovic MD Abrahamson EE Uz T Manev H Dekosky ST Increased 5-lipooxygenase imunoreactivity in hippocampus of patients with Alzheimer’ diseases J Histochem Cytochem 2008 56 1065 1073 10.1369/jhc.2008.951855 18678882 PMC2583907

[B5] Firuzi O Zhuo J Chinnici CM Wisniewski T Praticò D 5-lipoxygenase gene disruption reduces amyloid-β pathology in a mouse model of Alzheimer’s disease FASEB J 2008 22 1169 1178 17998412 10.1096/fj.07-9131.com PMC2698428

[B6] Diamant Z Timmers MC van der Veen H Friedman BS De Smet M Depré M Hilliard D Bel EH Sterk PJ The effect of MK-591, a novel 5-lipoxygenase-activating protein inhibitor, on leukotriene biosynthesis and allergen-induced airway responses in asthmatic subjects in vivo J Allergy Clin Immunol 1995 95 42 51 10.1016/S0091-6749(95)70151-6 7822663

[B7] Praticò D Uryu K Leight S Trojanowski J Lee V Increased lipid peroxidation precedes amyloid plaque formation in an animal model of Alzheimer’s disease J Neurosci 2001 21 12 4183 4187 11404403 10.1523/JNEUROSCI.21-12-04183.2001 PMC6762743

[B8] Yang H Zhuo J Chu J Chinnici C Praticò D Amelioration of the Alzheimer’s disease phenotype by absence of 12/15-lipoxygenase Biol Psych 2010 68 10 922 929 10.1016/j.biopsych.2010.04.010 20570249

[B9] Chu J Giannopoulos PF Ceballos-Diaz C Golde TE Praticò D Adeno-associated virus-mediated brain delivery of 5-lipoxygenase modulates the AD-like phenotype of APP mice Mol Neurodegener 2012 7 1 10.1186/1750-1326-7-1 22222029 PMC3277480

[B10] Chu J Praticò D 5-lipoxygenase as an endogenous modulator of amyloid beta formation in vivo Ann Neurol 2011 69 34 46 10.1002/ana.22234 21280074 PMC3051361

[B11] Kenneth JL Thomas DS Analysis of relative gene expression data using real time quantitative PCR and the 2-ΔΔCt method Methods 2002 25 402 408 10.1006/meth.2001.1262 11846609

[B12] Kawarabayashi T Younkin LH Saido TC Shoji M Hsiao Ashe K Younkin SG Age-dependent changes in brain, CSF, and plasma amyloid (beta) protein in the Tg2576 transgenic mouse model of Alzheimer’s disease J Neurosci 2001 21 2 372 381 11160418 10.1523/JNEUROSCI.21-02-00372.2001 PMC6763819

[B13] Leissring MA Farris W Chang AY Walsh DM Wu X Sunh X Frosch MP Selkoe DJ Enhanced proteolysis of beta-amyloid in APP transgenic mice prevents plaque formation, secondary pathology, and premature death Neuron 2003 40 1087 1093 10.1016/S0896-6273(03)00787-6 14687544

[B14] Guenette SY Mechanisms of Abeta clearance and catabolism Neuromolecular Med 2003 4 147 160 10.1385/NMM:4:3:147 14716023

[B15] Yao Y Chinnici C Tang H Trojanowski J Lee V Praticò D Brain inflammation and oxidative stress in a transgenic mouse model of Alzheimer-like brain amyloidosis J Neuroinflammation 2004 1 21 10.1186/1742-2094-1-21 15500684 PMC527877

[B16] Miller DK Gillard JW Vickers PJ Sadowski S Léveillé C Mancini JA Charleson P Dixon RA Ford-Hutchinson AW Fortin R Identification and isolation of a membrane protein necessary for leukotriene production Nature 1990 343 278 281 10.1038/343278a0 2300172

[B17] Dixon RAF Diehl RE Opas E Rands E Vickers PJ Evans JF Gillard JW Miller DK Requirement of a 5-lipoxygenase-activating protein for leukotriene synthesis Nature 1990 343 282 284 10.1038/343282a0 2300173

[B18] Evans JF Lévillé C Mancini JA Prasit P Thérien M Zamboni R Gauthier JY Fortin R Charleson P MacIntyre DE Luell S Bach TJ Meurer R Guay J Vickers PJ Rouzer CA Gillard JW Miller DK 5-lipoxygenase-activating protein is the target of a quinoline class of leukotriene synthesis inhibitors Mol Pharmacol 1991 40 22 27 1857337

[B19] Chu J Pratico D Pharmacological blockade of 5-lipoxygenase improves the amyloidotic phenotype of an Alzheimer’s disease transgenic mouse model Am J Pathol 2011 178 4 1762 1769 10.1016/j.ajpath.2010.12.032 21435457 PMC3078454

[B20] Ferguson AD McKeever BM Xu S Wisniewski D Miller DK Yamin TT Spencer RH Chu L Ujjainwalla F Cunningham BR Evans JF Becker JW Crystal structure of inhibitor-bound human 5-lipoxygenase-activating protein Science 2007 317 510 512 10.1126/science.1144346 17600184

[B21] Luo WJ Wang H Li H Kim BS Shah S Lee HJ Thinakaran G Kim TW Yu G Xu H PEN-2 and APH-1 coordinately regulate proteolytic processing of presenilin 1 J Biol Chem 2003 278 7850 7854 10.1074/jbc.C200648200 12522139

[B22] Wang R Zhang YW Sun P Liu R Zhang X Zhang X Xia K Xia J Xu H Zhang Z Transcriptional regulation of Pen-2, a key component of the γ-secretase complex, by CREB Mol Cell Biol 2006 26 1347 1354 10.1128/MCB.26.4.1347-1354.2006 16449647 PMC1367199

[B23] Chu J Zhuo JM Pratico D Transcriptional regulation of β-secretase-1 by 12/15-lipoxygenase results in enhanced amyloidogenesis and cognitive impairments Ann Neurol 2012 71 1 57 67 10.1002/ana.22625 22275252 PMC3270901

[B24] Hardy J Selkoe DJ The amyloid hypothesis of Alzheimer’s disease: progress and problems on the road to therapeutics Science 2002 297 353 356 10.1126/science.1072994 12130773

[B25] Wolfe MS Inhibition and modulation of γ-secretase for Alzheimer’s disease Neurotherapeutics 2008 5 391 398 10.1016/j.nurt.2008.05.010 18625450 PMC2572079

[B26] Geling A Steiner H Willem M Bally-Cuif L Haas C A γ-secretase inhibitor blocks Notch signaling in vivo and causes a severe neurogenic phenotype in zebrafish EMBO Rep 2002 3 688 694 10.1093/embo-reports/kvf124 12101103 PMC1084181

[B27] Searfoss GH Jordan WH Calligaro DO Galbreath EJ Schirtzinger LM Berridge BR Gao H Higgins MA May PC Ryan TP Adipsin, a biomarker of gastrointestinal toxicity mediated by a functional γ-secretase inhibitor J Biol Chem 2003 278 46107 46116 10.1074/jbc.M307757200 12949072

